# The Application of Brain Organoid Technology in Stroke Research: Challenges and Prospects

**DOI:** 10.3389/fncel.2021.646921

**Published:** 2021-06-21

**Authors:** Guini Song, Min Zhao, Hanmin Chen, Xiangyue Zhou, Cameron Lenahan, Yibo Ou, Yue He

**Affiliations:** ^1^Department of Neurology, Tongji Hospital, Tongji Medical College, Huazhong University of Science and Technology, Wuhan, China; ^2^Department of Neurosurgery, Tongji Hospital, Tongji Medical College, Huazhong University of Science and Technology, Wuhan, China; ^3^Department of Biomedical Sciences, Burrell College of Osteopathic Medicine, Las Cruces, NM, United States

**Keywords:** brain organoids, pluripotent stem cell, stroke, model, transplantation

## Abstract

Stroke is a neurological disease responsible for significant morbidity and disability worldwide. However, there remains a dearth of effective therapies. The failure of many therapies for stroke in clinical trials has promoted the development of human cell-based models, such as brain organoids. Brain organoids differ from pluripotent stem cells in that they recapitulate various key features of the human central nervous system (CNS) in three-dimensional (3D) space. Recent studies have demonstrated that brain organoids could serve as a new platform to study various neurological diseases. However, there are several limitations, such as the scarcity of glia and vasculature in organoids, which are important for studying stroke. Herein, we have summarized the application of brain organoid technology in stroke research, such as for modeling and transplantation purposes. We also discuss methods to overcome the limitations of brain organoid technology, as well as future prospects for its application in stroke research. Although there are many difficulties and challenges associated with brain organoid technology, it is clear that this approach will play a critical role in the future exploration of stroke treatment.

## Introduction

Stroke is a leading cause of disability and morbidity worldwide, and its prevalence has increased in recent years. Stroke causes substantial economic and social burdens that have serious impacts on the health and lives of people worldwide (Benjamin et al., 2018). Poststroke brain injury primarily results from energy metabolism disorders, cytotoxicity, neuroinflammatory responses, oxidative stress, and a series of pathophysiological changes (Khoshnam et al., 2017). However, there are few effective therapies for stroke. Tissue plasminogen activator (tPA) is used for the treatment of ischemic stroke but must be given within 4.5 h of stroke onset (Lozano et al., 2012). Although many agents have shown protective effects against stroke in preclinical studies and animal models, no obvious effects have been observed in clinical practice. This is related to the difference in brain structure and physiology between humans and rodents, which poses a challenge for stroke research and clinical translation (Chamorro et al., 2016).

Brain organoids are stem cell-derived three dimensional (3D) tissues that recapitulate brain organogenesis, biomechanical function, cytoarchitecture, and cell–cell interactions *in vitro*. They recapitulate early cerebral developmental events and present an opportunity to study and model various neurological diseases *in vitro*, such as neurodevelopmental diseases and psychiatric disorders (Lancaster et al., 2013; Wang P. et al., 2017). They can also recapitulate the *in vivo* brain microenvironment and are used to investigate synaptic connectivity, cell–tissue interaction, and cross-interaction within the immune system of the brain *in vitro* (Kelava and Lancaster, 2016; Qian et al., 2016). This provides a new opportunity for cerebrovascular disease studies. Brain organoids can be used for modeling stroke and for drug development for stroke treatments. Moreover, they could be used for regenerative rehabilitation for poststroke recovery. Human brain organoids are a promising tool for deepening our understanding of the development and physiology of the human brain and can be used to explore the pathogenesis of cerebrovascular diseases that occur due to acquired and inherited factors. As brain organoids are so similar to human brains, they could enhance the clinical translation of neuroprotective agents and are a promising approach for future stroke studies.

## Brain Organoid Techniques

With the development of cell culture and biomaterial technologies, there have been advances in 3D *in vitro* cerebral models. Brain organoids are 3D spherical cell aggregates, which possesses various characteristics resembling the physiology and structure of *in vivo* tissues. Brain organoids are mainly differentiated from human pluripotent stem cells (hPSCs), including human embryonic stem cells (ESCs) and human-induced pluripotent stem cells (iPSCs), which have self-renewing and self-organizing capabilities (Clevers, 2016; Dutta et al., 2017). The ability to self-organize results from specific intercellular signaling junctions and autonomous genetic programs, and the generation of brain organoids relates to cell fate and the innate self-organizing ability of neural cells (Turner et al., 2016). Intrinsic cell behavior leads to cell migration, polarization of the neuroepithelium, and cell subtype generation. iPSCs have the ability to differentiate into many cell types. Dual SMAD inhibition, Noggin, and SB431542 have been used to enhance neural fate choice and consistency with the environment for signaling exchange within the embryo (Chambers et al., 2009). High levels of Wnt signaling in the anterior neural tissue results in dorsalization of the cells and facilitates the differentiation of human neuroepithelium to cortical tissue, inhibiting ventral fates via Sonic hedgehog (SHH) antagonists to enhance cortical differentiation (van de Leemput et al., 2014). Studies have revealed that stem cells can be induced to differentiate into dopaminergic neurons (Nolbrant et al., 2017), cortical neurons (Shi et al., 2012a), motor neurons (Du et al., 2015), cholinergic neurons (Crompton et al., 2013), neural precursor cells (NPCs) (Nasonkin et al., 2009), and spinal cord neural stem cells (Kumamaru et al., 2018).

There are two methods to build 3D brain organoids: bottom–up and top–down approaches. The bottom–up approaches are related to the scaffolding architecture, which is promoted by biomaterial technology development (Tang-Schomer et al., 2014). Meanwhile, top–down approaches depend on the self-organization of hPSCs in the natural or artificial extracellular matrix (ECM), which consists of various highly controlled growth factors and molecules, such as the epidermal growth factor, Noggin, to guide cell differentiation along specific organ lineages (Lancaster and Knoblich, 2014b). Many organoids have been studied, including the kidney, retina, and liver (Lancaster and Knoblich, 2014b). Brain organoids were generated to study specific or multiple brain regions, such as the hippocampus, prefrontal cortex, cerebellum, and occipital lobe (Brawner et al., 2017). The most common approaches to culturing brain organoids are low-adhesion plate and large stirred bioreactor strategies. Low-adhesion plates are a useful tool to generate cell aggregates, such as embryoid bodies (EBs). Human ESCs are cultured in 96-well low-adhesion plates to generate cell aggregates (Eiraku et al., 2008). The generated clusters of neuroepithelial cells form rosette-like structures, showing features of the embryonic neural tube with preserved apical–basal polarity and containing distinct neuronal cells, such as ventricular radial glial cells and neuroepithelial stem cells, which recapitulate the developing brain (Shi et al., 2012b; Edri et al., 2015). However, the size of organoids cultured within low-adherence plates is limited by the lack of systems that deliver oxygen and nutrients to brain organoids. Therefore, large stirred bioreactors were invented to address the problems of oxygen and nutrient exchange. Brain organoids are encapsulated by ECM and suspended in bioreactors to grow (Lancaster et al., 2013). With the support of the ECM (Matrigel) and the spinning bioreactor, specific brain tissue-type organoids, including the forebrain, are produced in the presence of region-specific differentiation factors, such as SMAD inhibitors and brain-derived neurotrophic factor (BDNF) (Sloan et al., 2018).

Brain organoids include several cell types, such as neural stem cells (NSCs), NPCs, mature and immature neurons, and glial cells. They recapitulate the characteristics of neural connectivity in brain regions *in vivo* (Wang Z. et al., 2017; McCaughey-Chapman and Connor, 2018). Brain organoids can depict real-life developmental trajectories. Moreover, there is a correlation between the developmental stages of forebrain organoids and the human fetal brain (in particular, the prefrontal cortex) (Qian et al., 2016). The genetic profiles of organoids at 26–54 days correlate with those of fetal cortex neurogenesis at 8–9 gestation weeks (GW). Furthermore, the profiles of day 100 organoids resemble those of 17–35 GW cortical areas (Zecevic, 2004). Additionally, studies have revealed that gene expression programs in brain organoid cortex-like regions are similar to those of fetal tissue and organize into a structured cerebral cortex, and the major differences in gene expression seem to be a response to the culture environment (Camp et al., 2015).

Brain organoids have been used successfully to model neurodevelopmental diseases, such as abnormal cortical folding (Li et al., 2017). Lancaster used the cerebral cortex derived from human iPSCs, which were cultured in Matrigel and spinning bioreactors, to study cyclin-dependent kinase 5 regulatory subunit-associated protein 2 gene (CDK5RAP2) heterozygous nonsense mutation-related microcephaly. They found that neuroepithelial regions and organoid size were reduced and that the spindle orientation of radial glial cells had changed in the organoids (Lancaster et al., 2013). Qian et al. studied the effect of the Zika virus (ZIKV) on forebrain organoids derived from human iPSCs and found suppressed proliferation of neural progenitors. Moreover, they observed increased cell death, decreased neuronal layer thickness, and reduced organoid size (Qian et al., 2016). ZIKV attenuated cerebral cortex organoid growth via the Toll-like receptor 3 (TLR3)-mediated dysregulation of neurogenesis (Dang et al., 2016). Meanwhile, Garcez et al. found that ZIKV results in cerebral cortex organoids of a smaller size by inducing neural stem cell death and reducing neural progenitor cell growth (Garcez et al., 2016). ZIKV increased apoptosis in neural progenitors but reduced the proliferation zone and disrupted the cortical layers in cerebral cortex organoids (Cugola et al., 2016). Studies also revealed that the effect of cocaine on human brain development is related to the inhibition of neuroepithelial progenitor proliferation and reduction in cortical plate formation via cytochrome P450 3A5 (CYP3A5)-mediated cocaine oxidative metabolism in the neocortex (Lee et al., 2017). Modeling autism spectrum disorder with the dorsal telencephalon derived from human iPSCs, which overexpress the transcription factor forkhead box G1 (FOXG1), revealed enhanced synaptic maturation, increased progenitor cell proliferation, and overproduction of GABAergic inhibitory neurons (Mariani et al., 2015).

## Application of Brain Organoid Techniques for Stroke Modeling

Organoids derived from iPSCs are expected to fill the gap between humans and animal models. Currently, animal and human cell culture techniques remain the most common tools for fundamental pathophysiology research and preclinical drug testing in *in vitro* stroke studies. There are many cellular platforms that have been used in stroke studies, such as primary cells, cell lines, brain slices, organotypic cell cultures, ESCs, and iPSCs. Stroke can be modeled by ischemia-like conditions, in which the normal O_2_/CO_2_ medium is replaced with N_2_/CO_2_ medium. The cells are kept in a hypoxic chamber and are typically subjected to both oxygen and glucose deprivation (Holloway and Gavins, 2016). However, various studies have shown that two-dimensional (2D) cell cultures are not completely representative of the pathophysiological process of tissues *in vivo* after stroke (Duval et al., 2017). Compared with standard 2D cell cultures, 3D cultures are substantially different in terms of cell-ECM interactions, cellular mechanics, cellular behaviors, and nutrients as a result of the additional dimensionality. Due to the lack of complex vascular systems in 3D cultures, the oxygen and nutrients for culture are mainly obtained by diffusion. Compared with the rich oxygenation and nutrition environment for all cells in 2D monolayer culture, this restricted oxygenation and nutrition environment in 3D culture mimics the *in vivo* tissue microenvironment to a certain extent (Edmondson et al., 2014). Cells such as hippocampal neurons and astrocytes grown in 3D medium develop distinct phenotypes, have a higher neuron/astrocyte ratio and survive longer (Peretz et al., 2007). Meanwhile, these cells are more resistant to nutrient deprivation (East et al., 2009).

Many techniques have been used for 3D cell culture, such as hydrogels (alginate and collagen), synthetic microporous scaffolds (polystyrene scaffolds), bioreactors, and microfluidics (Duval et al., 2017). Scaffolds provide a biochemical composition and microarchitecture and are created from a plethora of materials (Lancaster et al., 2013). Mixtures of microfiber and nanofiber scaffolds made from polymers, such as polyethylene terephthalate and polylactic acid, produce structurally small pore sizes and increase cellular adhesion (Hejazian et al., 2012). Zhang et al. (2015b) investigated hollow channel-modified porous silk scaffolds to support the extensive vascularization process and found that vascularization was promoted *in vitro* by facilitating endothelial cell growth. Bioreactors have been used to assist 3D cell culture. Using bioreactors with perfusion, dynamic loading, and microfluidic actuation can accelerate the transportation of gas, nutrients, and wastes through 3D cellular scaffolds more effectively than using a natural diffusion approach (Yeatts et al., 2013). 3D-printed bioreactors are an economical method for organoid culture. Miniaturized spinning bioreactors have plastic lids that incorporate mini-stirrers attached to an electric motor rather than the large orbital shakers or spinning bioreactors found in tissue culture incubators (Qian et al., 2016). Microfluidic devices are usually derived from polymers, such as poly(dimethylsiloxane) (PDMS), and their structure allows the formation of organs-on-chip, which mimics the function of organoids (Bhatia and Ingber, 2014).

Human somatic cells, such as skin fibroblasts and blood cells, can be reprogrammed into iPSCs *in vitro* and display unlimited self-renewal (Yu et al., 2007). iPSCs have been studied in models of stroke related to genetic factors, such as the Marfan syndrome (Granata et al., 2017), cerebral autosomal dominant arteriopathy with subcortical infarcts and leukoencephalopathy (CADASIL) (Kelleher et al., 2019; Ling et al., 2019), moyamoya disease (Tokairin et al., 2020), and cerebral cavernous malformation (CCM) (Molina et al., 2020). Many studies have explored the effects of hypoxia and heme on brain organoids. Harbuzariu et al. (Harbuzariu et al., 2019) studied the effects of heme in cerebral malaria using a 3D cortical organoid system. They found heme-induced cell apoptosis and structural changes. Moreover, they revealed that neuregulin-1 (NRG-1) protects cells against heme in organoids, providing a potential mechanism for modeling and studying hemorrhagic stroke. The negative effect of hypoxia on human brain development, such as the development of the dorsal brain, oligodendrocytes, and neuronal progenitors, has also been studied using brain organoids, and the effects could be extenuated by the use of minocycline (Boisvert et al., 2019). Paşca et al. (Paşca et al., 2019) found a reduction in intermediate progenitors in human brain organoids cultured in hypoxic conditions, the expansion of the human cerebral cortex, and an increased unfolded protein response. One study showed that transient hypoxia caused the apoptosis of outer radial glia and decreased the differentiation of immature neurons in brain organoids. Daviaud et al. (2019) and Kim et al. (2021) developed brain organoids from induced neural stem cells (iNSCs), which are directly derived from human somatic cells but do not involve embryoid bodies or neural induction. Brain organoids were under hypoxic conditions consisting of 1% oxygen for 48 h on day 84 and were then transferred to normoxic conditions consisting of 21% oxygen and 5% CO_2_ for 24 h (for reoxygenation). Oxygen deprivation resulted in injury and a decrease in the size of the brain organoids. Meanwhile, reoxygenation could not reverse the hypoxic effects on the ventricular zone and cortical plate layers. Reoxygenation induced cell proliferation but could not restore neuronal maturation.

Blood–brain barrier (BBB) organoids have also been studied. BBB organoids are spontaneously assembled by human primary brain endothelial cells and pericytes in coculture under low-adherence conditions and resemble the integrity characteristic of the BBB because each of the cell types directly interact with one another within the spheroid (Cho et al., 2017; Bhalerao et al., 2020). The spheroid core mainly consists of astrocytes, while the surface of the spheroids consists of brain endothelial cells and pericytes that regulate the transport of molecules. The organoids highly express tight junction proteins and receptor-mediated transcytosis of angiopep-2, further recapitulating the characteristics of the BBB (Cho et al., 2017). Nzou and his colleagues (Nzou et al., 2020) developed a 3D neurovascular unit organoid using the hanging drop culture method, consisting of neurons, oligodendrocytes, astrocytes, microglia, human brain microvascular endothelial cells, and pericytes; when cultured under hypoxic conditions, these organoids recapitulate the characteristics of BBB hypoxia-mediated dysfunction and show increased permeability, inflammation, and oxidative stress. The anti-inflammatory agents 2-arachidonoyl glycerol and secoisolariciresinol diglucoside protect against hypoxia-induced inflammation in the organoids. This provides support for using organoids to study drug transport through the BBB and demonstrates the utility of BBB organoids for drug development and candidate screening.

Brain organoids have been used to model brain malformations caused by infectious diseases, genetic deficits, neurodegenerative diseases, and psychiatric disorders, and they recapitulate disease-relevant phenotypes to allow for the further study of their characteristics (Lancaster et al., 2013; Garcez et al., 2016; Wang P. et al., 2017; Papaspyropoulos et al., 2020). However, the lack of a vascular system and microglia in brain organoids remains a limitation for stroke modeling (Di Lullo and Kriegstein, 2017; Chen et al., 2019). Blood flow, vascular cell-derived factors, the composition of blood, and microglia play important roles in the pathophysiological changes in the brain resulting from stroke, and the presence of a vascular system would be better for stroke modeling. Stroke is primarily a disease of the elderly, and brain organoids that mimic human fetal brain development may not accurately recapitulate stroke-relevant phenotypes. Due to the development of culture technology, brain organoids can be cultured for extended periods, and these long-term cultures can be used to model late-onset diseases beyond the developmental stage, such as neurodegenerative diseases (Lancaster and Knoblich, 2014b). Future stroke modeling requires studies of pathophysiological changes in brain organoids with vascular systems and microglia after 6 months or longer in culture. Although studies of 3D organoid models of stroke are currently limited, the development of organoid strategies has the potential to allow researchers to study more naturally organized 3D cell models in stroke research.

## Transplantation of 3D Brain Organoid Tissue for Poststroke Repair

Regenerative medicine is important for nerve regeneration after stroke. Stem cell transplantation studies support the use of regenerative therapy for repairing injured tissues after stroke. Stem cells have synergistic mechanisms that account for the beneficial effects observed in experimental stroke models, such as neurogenesis, neuroprotection, angiogenesis, trophic factor production, new cell generation, and immune response modulation (Chau et al., 2014; Cefalo et al., 2016). The transplantation of mesenchymal stem cells (MSCs) or pluripotent stem cells has been investigated in many preclinical studies and these cells have shown promise for use in rehabilitative stroke therapy (Takagi et al., 2017; Deng et al., 2019). Transplanted NSCs also increase neural repair and promote functional recovery after stroke. Neural progenitor cells have the ability to differentiate into neuronal and glial cell types and have been transplanted as a cell replacement therapy. The ability of iPSCs to fully differentiate makes them valuable candidates for cell transplantation therapies (Yu et al., 2007). However, safety is an important issue in preclinical cell therapy for stroke. iPSCs are generated using a retroviral vector to transfer oncogenes (Oct4, Sox2, Klf4, and c-Myc) into human fibroblasts, which leads to a significant risk for tumorigenesis (Takahashi et al., 2007). Therefore, developing an optimal method to generate iPSC-NSCs is extremely important. Recent reprogramming of somatic cells using an adenovirus-mediated gene delivery system has improved the generation of iPSCs, and other techniques, such as transducing without the Myc retrovirus, can remove the risk of oncogene integration, thus, reducing the incidence of tumorigenicity (Nakagawa et al., 2008; Okita et al., 2008). Although most reported clinical trials to date are preliminary and have an insufficient number of enrolled patients, a negative effect was reported in only one out of 43 published clinical trials in which patients experienced seizures and motor function aggravation after transplantation with xenogeneic fetal porcine cells. This indicates that stem cell transplantation appears to be safe in most cases (Kawabori et al., 2020).

**FIGURE 1:**
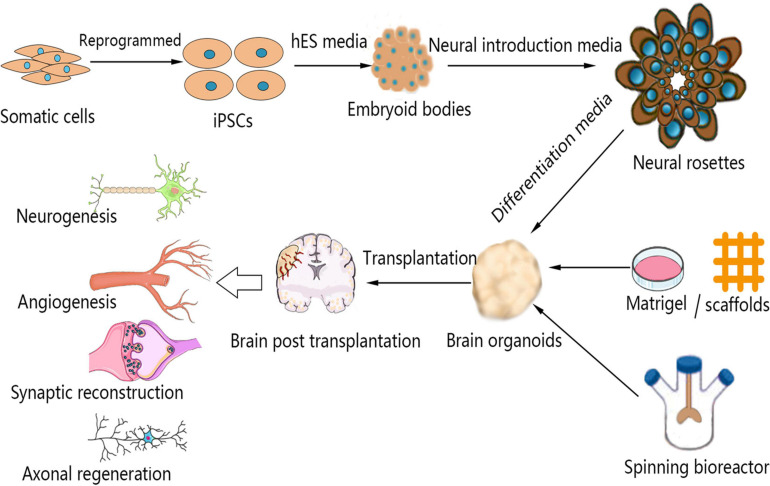
Schematic representation of the brain organoid culture process and the mechanism by which brain organoid transplantation enhances neural regeneration in stroke. Somatic cells can be reprogrammed to produce iPSCs, which can be used for the culture of brain organoids. The 3D aggregation of iPSCs under conditions of neural induction molecules causes the formation of neural rosettes. These structures can be suspended in Matrigel or scaffolds to develop into brain organoids in a spinning bioreactor. Brain organoids are transplanted into injured brains after stroke. Transplanted brain organoids are vascularized by host endothelial cells, and the brain infarct volume is reduced by cell migration and replacement. Moreover, brain organoid transplantation enhances neurogenesis, angiogenesis, synaptic reconstruction, and axonal regeneration after stroke. iPSCs, induced pluripotent stem cells.

Although diverse cell types are damaged by stroke, only a single type of NSC or neural cell is typically used in cell transplantation studies to repair brain injury, which may be insufficient to regenerate all damaged cell types (Wang and Miao, 2015; Wei L. et al., 2017). Brain organoids consist of abundant neural cell types and can be a rich source of cells for transplantation and regeneration to repair brain injury (Figure 1). 3D brain organoid tissue transplants can consist of a complete microenvironment, including neuronal progenitors, mature neurons, astrocytes, and oligodendrocytes (Lancaster and Knoblich, 2014a; Kim et al., 2019). The main studies on brain organoid transplantation are listed in Table 1. One study revealed that hNSCs were dispersed in a 3D artificial ECM and differentiate *in vitro.* Next, grafting the cells into rat pups at postnatal day 14 resulted in the *in vivo* formation of brain organoids; intrinsic factors directed the organization of brain organoids and promoted the infiltration of host blood vessels into the organoids (Basuodan et al., 2018). Cultured ESCs and iPSCs formed six-layer identities and were transplanted into the mouse brain, where they established specific axonal connectivity and functional synapses after differentiation. Transplanted human cortical neurons differentiate progressively over several months *in vivo*, establishing connectivity with the host circuitry and offering opportunities for brain repair (Espuny-Camacho et al., 2013). The human iPSC-derived neuronal networks can retain physiological activities and are structurally integrated into the rat forebrain, participating in neural network activity via functional synaptic integration *in vivo* (Yin et al., 2019).

**TABLE 1:** Main studies in brain organoid transplantation.

Transplanted organoids	Cell type derived	Extracellular scaffold	Receiver	Evaluation time post implantation	Blood supply	Immunosuppressive treatment	Main finding	References
Brain organoids at 47–48 DIV	hESCs hiPSCs	Matrigel	P8–P10 CD1 mice	2 and 4 weeks	Host brain	None	Increased cell survival and neuronal differentiation	Daviaud et al., 2018
Brain organoids at 40–50 DIV	hESCs	Matrigel	NOD/SCID mice	0.5–8 months	Host brain	None	Increased neuronal differentiation and maturation; developed functional synaptic connectivity and neuronal activity between grafted and host brain	Mansour et al., 2018
Brain organoids at 55 DIV	hESCs	Matrigel	Sprague–Dawley rats	4 weeks	Host brain	Cyclosporine A	Reduced cerebral infarct volume; enhance axonal regeneration and synaptic reconstruction	Wang S. N. et al., 2020
Brain organoids at 55 and 85 DIV	hESCs	Matrigel	Sprague–Dawley rats	8 weeks	Host brain	Cyclosporine A	Increased neurogenesis and cell survival; improved motor function and reduced brain injury; 55-day brain organoids are better donors than that of 85-day;	Wang Z. et al., 2020
Brain organoids at 42 and 70 DIV	hESCs	ND	SCID mice; cynomolgus monkeys	12 weeks	Host brain	None; tacrolimus hydrate	Extended axons along the host corticospinal tract; 42-day organoids caused graft overgrowth after transplantation	Kitahara et al., 2020
Brain organoids at 40–50 DIV	hESCs hiPSCs	ND	SCID mice	1–5 months	ND	None	Increase the startle fear response of host; showing subcortical projection establishment	Dong et al., 2020

*DIV, days *in vitro*; ND, not described.*

One study showed that when brain organoids are grafted onto mouse cortex lesions, there is increased cell survival and better neuronal differentiation compared with that observed after neural stem cell transplantation (Daviaud et al., 2018). Human brain organoids that are grafted into immunodeficient mouse brains are vascularized by endothelial cells from the host brain. These organoids showed neuronal differentiation and maturation, and functional synaptic connectivity and neuronal activity developed between the transplanted brain organoids and the host brain, which was confirmed by electrophysiological recording and optogenetics (Mansour et al., 2018). Another approach to analyzing synapses and visualizing synaptogenesis in the graft-to-host brain can be performed through confocal microscopy (Yakoub and Sadek, 2019). Wang et al. transplanted brain organoids at 55 days into rats with middle cerebral artery occlusion (MCAO). They found that the cerebral infarct volume was reduced and that cells from brain organoids migrate into various brain regions, in which they enhance axonal regeneration and synaptic reconstruction after stroke. However, brain organoid transplantation has no effect on cell apoptosis or neuroinflammation in the ipsilateral cortex, showing the compatibility between brain organoids and the host brain (Wang S. N. et al., 2020). Brain organoids are diverse in neural cell types and cell numbers at different stages. One study showed that 55-day brain organoids are a better transplantation donor than 85-day organoids, with more neurogenesis and increased cell survival after transplantation. Additionally, there was improved motor function and reduced brain injury in a rat traumatic brain injury model. Meanwhile, transplanted brain organoids were successfully vascularized (Wang Z. et al., 2020). This study implies that brain organoids with a greater number of neuronal progenitors could be more beneficial for nerve regeneration.

Kitahara et al. (Kitahara et al., 2020) found that transplantation of 6-week organoids caused graft overgrowth in 7-day-old mice; these organoids contained a larger number of proliferative cells and extended more axons along the host corticospinal tract than 10-week organoids. Meanwhile, delayed transplantation of 10-week organoids after lesioning can extend more axons in 6-week-old mice than transplantation without delay, and 10-week organoids had survival in the monkey brain. Another study revealed that centimeter-long human axon tracts, generated and cultured in hydrogel microcolumns, which permits physical manipulation and includes discrete cellular regions around axon tracts, were transplantable. This transplantation can be performed with no need for long-range axonal regeneration (Cullen et al., 2019). Additionally, small human brain organoids that have been grafted into the mouse have survived and acquired electrophysiological maturity. However, mice that received transplants with brain organoids demonstrated an increase in the startle fear response, showing subcortical projection establishment (Dong et al., 2020). Large animals have a brain with a gray-to-white matter ratio approximating that of humans. The cerebral blood supply and cerebrovascular architecture often also resemble those of humans, making these animal models appropriate for physiological monitoring after brain organoid transplantation (Herrmann et al., 2019).

Many potential imaging strategies, such as magnetic resonance imaging (MRI) and positron emission tomography (PET), can be used to monitor engraftment clinically. Engrafted tissues demonstrated high intensity on T2-weighted MR images and low intensity on T1-weighted MR images (Kitahara et al., 2020). Meanwhile, supraparamagnetic iron oxide (SPIO) particles are approved by the FDA for use as contrast agents for cell labeling and *in vivo* tracking, which improves the 3D spatial resolution of MRI (Manley and Steinberg, 2012). Moreover, MRI using SPIO has been used for human clinical research on stem cell transplantation. Spinal cord injury patients were transplanted with SPIO-labeled autologous bone marrow CD34 + cells, and some of them showed a persistent hypointense signal around the lesion site on MRI (Callera and de Melo, 2007). Zhang et al. used spatiotemporal PET imaging to observe dynamic metabolic changes after iPSC transplantation in stroke patients and found that iPSCs may differentiate into a more specific cell type before improving recovery from cerebral injury (Zhang et al., 2015a). Moreover, cerebral blood flow can be monitored via simultaneous [^15^O] H_2_O PET/MRI after stroke (Werner et al., 2015). This provides a method to monitor structural repair and metabolic activity after brain organoid transplantation and engraftment in the clinical setting.

Compared with stem cells, 3D organoids differ in cell migration capacity, which is related to the complicated cell–matrix interactions and cell interactions (Gjorevski et al., 2015). Cells in 3D organoids are surrounded by matrix substrate, which hinders cell migration and substantially influences the migration speed. The speed of migration in 2D and 3D cultures relates to matrix substrates. Fibroblasts migrate faster in 3D cell culture than in 2D culture on fibrin, collagen, and cell-derived matrix. Additionally, migration was faster in 2D cell culture with basement membrane extract (Hakkinen et al., 2011). Brain organoids can integrate with the host tissue due to their self-renewal and self-organization after transplantation, which maintains the organoid cells in the brain (Wei N. et al., 2017). 3D scaffolds can provide an environment for cell migration in stroke repair. Damaged areas can be scanned with MRI to design the shape of scaffolds for 3D printing to meet injury-specific requirements pertaining to size, shape, and cell content for individual transplantation (Fu et al., 2017). There are many questions that warrant further study in the regenerative therapy of stroke, such as mechanisms of action and optimal time of brain organoid transplantation. The challenge of transplanting brain organoids into the injured brain is an enormous obstacle. It was observed that 6-week organoids caused graft overgrowth after transplantation in mice (Kitahara et al., 2020). It is possible that promoting brain organoids to become more mature before transplantation and decreasing the volume of grafted organoids could reduce the risk for graft overgrowth in the injured brain and improve transplantation safety. With the development of transplantation technologies, it is likely that brain organoids will play an important role in stroke treatment and recovery.

## Limitations of Brain Organoid Technology

It is obvious that the generation of 3D brain organoids offers a complex model system that presents the opportunity to model various neurological diseases. There are still limitations in the current brain organoid technologies. Unlike monolayer culture systems, the culture systems used to generate 3D brain organoids from iPSCs are diverse. After brain organoids have been cultured, there are many differences between *in vitro* cultured organoids and *in vivo* tissues. Some non-neural cells are very important for the development of the local environment in the neural system; for example, endothelial cells differentiated from mesoderm-derived endothelial progenitors, microglia differentiated from yolk sac-derived macrophages that develop in the local brain environment, and pericytes from neural crest cells, which are important for the vascular systems (Balikov et al., 2020). Therefore, cells in brain organoids lack vascular systems as a result of the restricted culture techniques, which is the main challenge for stroke modeling and transplantation. Compared with brain organoids, cells *in vivo* are surrounded by capillaries that supply nutrients and oxygen (Wörsdörfer et al., 2019). The supply of gas and nutrients to brain organoids mainly depends on simple diffusion from the medium, which causes a number of organoid cells to undergo apoptosis because of a lack of oxygen and nutrients after a long culture period. Therefore, it is necessary to establish a circulatory system for long-term *in vitro* culture, which is important for stroke studies using brain organoids.

In addition to the lack of a vascular system, stromal components, including microglia, which are closely related to poststroke pathophysiological changes, are absent from current brain organoids, substantially limiting their application in stroke modeling and research (Ormel et al., 2018). There are various types of non-neural cells in the brain, such as microglia, hematopoietic cells, and meninges. Current protocols in brain organoid culture induce a neuroectodermal fate in the embryoid body, causing a deficiency in non-neural cells. Thus, brain organoids cannot model neurological diseases that involve interactions between neural cells and non-neural cells, especially stroke. The current culture protocols for brain organoids mainly depend on the self-organization capability of neuroepithelial cells. However, the size of the embryoid body and environmental factors are hard to control in experimental culture systems. Meanwhile, the underlying mechanisms by which stem cells self-organize remain unknown. This causes high variability between brain organoids, and it is difficult to better control the specific cell types and cell organization process (Lancaster and Knoblich, 2014a). Brain organoids cultured *in vitro* are different in size and shape, and the positions of the brain regions within each organoid also differ. The high heterogeneity of brain organoids makes it difficult to use organoids for fine quantitative and large-scale unbiased studies. Another limitation is the maturation of brain organoids *in vitro*. Most brain organoids approach complete maturation when transplanted *in vivo* (Mansour et al., 2018). This poses challenges for the application of brain organoids for stroke.

## Future Application Prospects of Brain Organoid Technology

**FIGURE 2:**
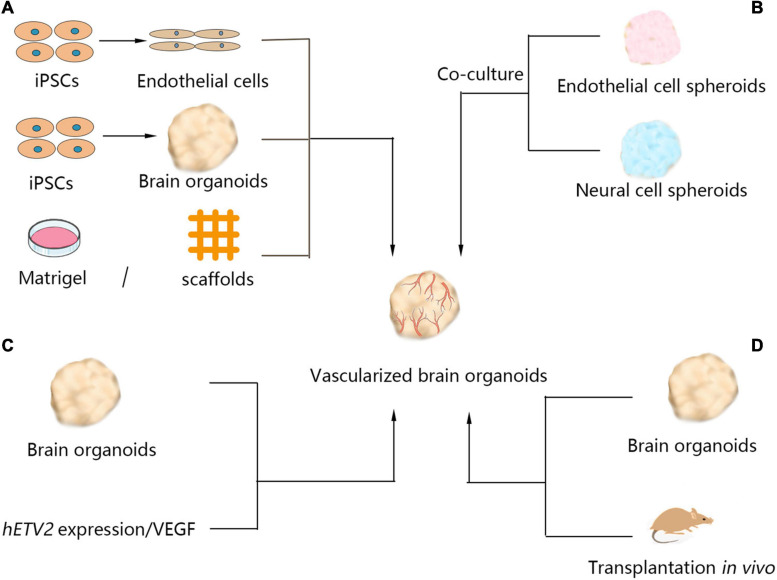
Currently available methods of brain organoid vascularization. Four methods have been used for vascularization of organoids. **(A)** Brain organoids cocultured with endothelial cells dispersed in the extracellular matrix, such as Matrigel or scaffolds. **(B)** Neural cell spheroids cocultured with endothelial cell spheroids generate brain organoids with vascular systems. **(C)** Induction of *hETV2* expression or treatment of brain organoids with VEGF in the culture process also produced vascular systems in brain organoids. **(D)** Brain organoids were transplanted into hosts, such as mice, and the grafts were vascularized by the migration of host endothelial cells. iPSCs, induced pluripotent stem cells; VEGF, vascular endothelial growth factor; *hETV2*, human ETS variant 2.

Various methods have been developed to vascularize brain organoids *in vitro* (listed in Figure 2). In brain organoids, vascular network recruitment is important for neurogenesis (Bjornsson et al., 2015). Vascular endothelial cells are mainly differentiated from the mesodermal lineage, and the absence of cells derived from the mesodermal lineage in the brain organoid culture procedure causes a lack of vascularization. Coculturing with endothelial cells is one method to induce vascularization in brain organoids. Pham et al. (Pham et al., 2018) studied a human vascularized brain organoid model by culturing organoids in Matrigel containing endothelial cells. Although the remaining vascular systems were not perfusable, and some areas of the brain organoid remained avascular, this study showed that vascularization of brain organoids is technically achievable. Another approach to vascularize brain organoids is to fuse mesodermal precursor cells (MPCs) and brain organoids in culture, which has been shown to result in a blood vessel-like ultrastructure with a high plasticity in brain organoids, including a basement membrane, endothelial cell-cell junctions, and luminal caveolae. These organoids are responsive to hypoxia, and the vessels in tumor organoids connect to host vessels after grafting (Wörsdörfer et al., 2019). Fusing endothelial cell (iEC) spheroids, cortical neural precursor cell (iNSC) spheroids, and mesenchymal stem cells into hybrid neurovascular spheroids can also induce vascular development (Song et al., 2019). Moreover, hESC differentiation with vascular endothelial growth factor (VEGF) could generate blood vessels in cultured brain organoids (Ham et al., 2020). Brain organoids differentiated from wild-type hESCs and engineered hESCs that ectopically express ETS variant 2 (*ETV2*) contain endothelial cells and form vascular-like networks (Cakir et al., 2019).

Additionally, synthetic scaffold bioprinting is a method to promote the vascularization of brain organoids. Using bioprinting technology, human tissues with thick vascular networks were generated (Kolesky et al., 2016). Schwartz et al. established 3D neural constructs with microglia and vascular networks by combining hESC-derived neural progenitors, microglia precursors, endothelial cells, and mesenchymal stem cells on chemically defined polyethylene glycol hydrogels (Schwartz et al., 2015). These brain organoids with vascularization have increased expression of tight junctions and transendothelial electrical resistance, but further research is warranted to develop functional vascular networks in brain organoids (Auger et al., 2013). Meanwhile, materials that can break down over a planned period of time have been used to control network geometry and provide room for cell growth, such as rigid 3D filament networks of carbohydrate glass and poly(N-isopropylacrylamide) microfiber gelatin hydrogels (Miller et al., 2012; Lee et al., 2016). Balikov et al. (2020) proposed using technologies that combine a top–down approach with a bottom–up approach to achieve the vascularization of brain organoids. Brain organoids can be differentiated in a scaffold with microchannels (bottom–up culture), and then stromal and endothelial cells can be seeded in microchannels to form vascular systems (top–down approach). This approach is needed to prevent the dissolution of microchannel architectures in organoid culture.

One study found that brain organoids cultured without dual-SMAD inhibition contain mesodermal-derived microglial cells that respond to inflammatory stimulation in a way that closely mimics the response of adult microglial cells (Ormel et al., 2018). In addition, iPSCs can be directed to differentiate into microglia in well-plate cultures through hematopoiesis (Muffat et al., 2016; Douvaras et al., 2017). Coculture with 2D-differentiated microglial cells has been employed to explore the interactions between microglial cells and neural cells in brain organoids (Lin et al., 2018). However, after prolonged brain residence, bone marrow-derived macrophages still differ from host microglia in their transcriptomic and chromatin landscape (Shemer et al., 2018). Thus, the brain organoid microenvironment could possibly affect endogenous or exogenous microglia, and more investigations are needed to assess approaches to integrate microglia into organoids.

Studies have also been undertaken to improve the homogeneity of organoids and facilitate better control of the growth of brain organoids. Optimal organoid culture approaches can help enhance reproducibility and reduce heterogeneity *in vitro* (Qian et al., 2016). Additionally, 3D bioprinting technology is expected to offer a new approach to organoid culture systems. 3D bioprinting can assist in manipulating the size, shape, architecture, and tissue-specific composition of the organoids (Vijayavenkataraman et al., 2018). Moreover, one study showed that Notch signaling inhibition can prevent cell proliferation and induce terminal differentiation, which could facilitate brain organoid maturation (Nakano et al., 2012). Shortened telomeres cause age-associated phenotypes in hPSC-derived midbrain dopamine neurons. Therefore, pharmacological inhibition of telomerase in hPSC during early differentiation can increase age-associated markers and potential late-onset disease-associated phenotypes (Vera et al., 2016). Clinical studies of brain organoids should emphasize safety rather than confirming efficacy in early preclinical research. It is necessary to conduct exploratory research in standard stroke models, such as permanent middle cerebral artery occlusion. Meanwhile, large animal models allow more precise testing of brain organoid delivery techniques and allow us to study the dose of transplantation for human clinical trials; therefore, large animal models are recommended for preclinical studies. Issues related to drug–organoid interactions need to be considered. It is important for patients with injured brains to undergo advanced safety assessments of their immune response, infarct location and size, and medication use (Boltze et al., 2019). 3D corticomotor assembloids, which are assembled by organoids resembling the cortex, hindbrain/spinal cord, or human skeletal muscle spheroids, demonstrated a functional circuit formed by the self-assembly capacity of 3D cultures (Andersen et al., 2020). In future approaches, human brain organoids could be combined with a blood vessel organoid system to be used for stroke modeling and clinical transplantation.

## Conclusion

Brain organoids mimic many key characteristics of the normal brain that can be implemented to study various diseases and can function to supplement traditional animal and cell research methods. Moreover, brain organoids can recapitulate diseases that are difficult to model with current technologies. Brain organoids could represent a way to study stroke and promote neural regeneration. There are many limitations in using brain organoids for stroke modeling and transplantation, such as a lack of vascularization and microglial cells. Significant research must be conducted on brain organoids to develop stroke models and to promote neural regeneration with brain organoid transplantation.

## Author Contributions

GS and YH conceived the main outline. GS wrote the manuscript. MZ and HC made the table and figures. XZ, YO, and CL took charge of the manuscript revisions in English. All authors read and approved to submit and publish the manuscript.

## Conflict of Interest

The authors declare that the research was conducted in the absence of any commercial or financial relationships that could be construed as a potential conflict of interest.
